# Clinical Validation of Imaging Biomarkers in Mycosis Fungoides

**DOI:** 10.1111/exd.70236

**Published:** 2026-03-11

**Authors:** Selinde S. Wind, Elise S. M. Beljaards, Rianne Rijneveld, Lisa Bruijnincx, Tessa Niemeyer‐van der Kolk, Manon A. A. Jansen, Yalcin Yavuz, Marieke de Kam, Jacobus Burggraaf, Naomi Klarenbeek, Jacobus Bosch, Koen D. Quint, Maarten H. Vermeer, Robert Rissmann, Next‐Generation ImmunoDermatology Consortium (NGID), Rob Vreeken, Eva Cuypers, Sylvia van Beugen, Antoinette van Laarhoven, Boudewijn Lelieveldt, Abdoel El Ghalbzouri, Marieke Seyger, Juul van den Reek, Ellen van den Bogaard, Elke de Jong, Peter van den Broek, Bernd Arents, Ton de Leeuw, Tom Ederveen, Peter Bram ’t Hoen, Annemarie Sluijmers, Bernd Arents, Mieke de Leeuw, Ton de Leeuw, Peter van den Broek, Els van der Pool, Ellen Stijl‐’t Hart, Lisanne Wezendonk, Douwe Vellinga, Stephan Weidinger, Sandrine Dubrak, Yang Li, Maurice Steensel, Martina Marchetti‐Deschmann

**Affiliations:** ^1^ Centre for Human Drug Research Leiden the Netherlands; ^2^ Department of Dermatology Leiden University Medical Center Leiden the Netherlands; ^3^ Leiden Academic Centre for Drug Research Leiden the Netherlands

**Keywords:** biomarker, CAILS, cutaneous T‐cell lymphoma, imaging, mycosis fungoides, validation

## Abstract

The composite index lesion severity (CAILS) score is used to monitor disease and therapeutic response in mycosis fungoides (MF), but is limited by interobserver variability and low sensitivity. Emerging imaging techniques, such as multispectral imaging (MSI), colourimetry and laser speckle contrast imaging (LSCI), offer objective alternatives for quantifying CAILS parameters. The aim of this study was to evaluate non‐invasive imaging modalities for objective and reliable quantification of disease extent in MF. Sixty‐six participants were enrolled in two prospective studies: a cross‐sectional discovery cohort to assess baseline characteristics of 35 MF patients (IA–IVB) and 10 healthy controls using CAILS and MSI, and a longitudinal confirmation cohort including 21 early‐stage MF patients (IA–IIA) treated with chlormethine gel 0.016% for 16 weeks, in whom lesional and non‐lesional skin were assessed using CAILS, MSI, colourimetry and LSCI at multiple time points. Candidate biomarkers were required to meet five clinical validation criteria: disease discrimination, repeatability, treatment responsiveness, correlation with CAILS and patient acceptability. In the discovery cohort, MSI detected significant differences in erythema, pigmentation, elevation and desquamation between healthy, non‐lesional and lesional skin. In the confirmation cohort, four candidate biomarkers met all validation criteria: MSI CIELAB a*, MSI average haemoglobin, and colourimetry CIELAB a* (DSMIII) for quantifying erythema, and MSI individual typology angle (ITA) for pigmentation. These biomarkers reliably discriminated lesional from non‐lesional skin (*p* ≤ 0.001), showed strong test–retest reliability (CV < 10%, ICC > 0.84), detected treatment effects, showed moderate concordance with CAILS, and were associated with low patient burden (mean 3.4/100). These findings show that MSI‐ and colourimetry‐derived biomarkers can objectively monitor disease extent in MF and complement existing clinical assessments.

## 1 Introduction

Mycosis fungoides is the most common subtype of cutaneous T‐cell lymphoma (CTCL) and is clinically characterised by heterogeneous inflammatory skin lesions, ranging from erythematosquamous patches to indurated plaques in early stages (IA–IIA) [1, 2]. Approximately 30% of patients progress to a more aggressive tumour stage (IIB), with 10‐year survival declining significantly from 83% (Stage IB) to 42% [3].

Treatment depends on disease stage, with skin‐directed therapies such as topical chlormethine used in early stages [4], and more intensive systemic treatments reserved for advanced disease [5].

Accurate monitoring of disease activity and treatment response is crucial for therapeutic decisions. However, this remains challenging due to the heterogeneous presentation of MF and the lack of standardised, objective assessment tools [6].

Scoring systems, such as the Composite Assessment of Index Lesion Severity (CAILS) for individual lesion severity and modified Severity‐Weighted Assessment Tool (mSWAT) for overall disease severity, are currently used to monitor MF. However, these scores are limited by low sensitivity and substantial interobserver bias, particularly the CAILS score [7, 8]. The inclusion of both hypo‐ and hyperpigmentation in a single subscore and the difficulty in distinguishing patches from thin plaques or thick plaques from tumours further compromise its reliability [9, 10]. Notably, the CAILS score often underestimates clinical severity by assigning lower severity scores to small tumours than to large, scaly plaques, despite the prognostic importance of tumour formation [5, 11, 12]. These limitations underscore the need for more objective and reproducible assessment methods.

Recent studies have explored promising non‐invasive imaging tools for monitoring CTCL, such as dermoscopy, reflectance confocal microscopy, high‐frequency ultrasound and line‐field confocal optical coherence tomography. However, these modalities lack the reproducibility required for clinical validation and broader implementation [13, 14, 15, 16, 17, 18, 19, 20, 21, 22].

This study aims to support personalised care in CTCL by validating novel non‐invasive imaging biomarkers for objective and reproducible monitoring of disease activity and response to chlormethine gel therapy, using Kruizenga's methodology [23] (Figure S1), consistent with established clinical validation standards [24, 25, 26] (Figure S2). By employing advanced imaging techniques, including multispectral imaging (MSI), colourimetry and laser speckle contrast imaging (LSCI), we seek to overcome limitations of current clinical scoring systems, ultimately enabling distinction between responders and non‐responders.

## 2 Materials and Methods

### 2.1 Study Design

A prospective, observational, cross‐sectional study was conducted to identify candidate non‐invasive imaging biomarkers by comparing baseline characteristics of healthy controls to non‐lesional and lesional skin in patients (discovery cohort). An exploratory, open‐label, observational study followed to validate the candidate biomarkers by correlating findings with physician‐assessed severity scores during topical chlormethine treatment (confirmation cohort). Both studies were conducted at the Centre for Human Drug Research in Leiden, The Netherlands, under supervision of the Leiden University Medical Centre, from October 2021 to August 2023. The studies adhered to the Declaration of Helsinki and were approved by an independent medical ethics committee ‘Medisch‐Ethische Toetsingscommisie van de Stichting Beoordeling Ethiek Biomedisch onderzoek’ (Assen, The Netherlands). Ethical approval and written informed consent were obtained for all participants. The protocol for the discovery cohort is registered in the ClinicalTrials.gov database (NCT05827107), and the protocol for the confirmation cohort in the databases of EudraCT (2021‐001576‐41) and ClinicalTrials.gov (NCT05303480). The imaging findings presented are part of a multimodal exploratory study investigating diverse research methodologies to identify biomarkers to enhance both the identification of MF and the monitoring of therapeutic responses [27].

### 2.2 Patients and Treatment

In the discovery cohort, patients with a confirmed histopathological diagnosis of MF (Stage IA–IVB) were included if they were over 18 years old and presented with at least one patch, plaque, or tumour lesion. Patients visited the study site for one non‐interventional visit at baseline.

In the confirmation cohort, patients aged 18–75 with a histopathological diagnosis of early‐stage MF (IA‐IIA) were included if they had at least one patch or plaque lesion with a diameter ≥ 6 cm. After a washout period for topical and systemic CTCL treatments, patients had two non‐interventional visits at Weeks −6 and 0. At Week −6, a representative target lesion was selected and mapped for consistent scoring. After training on Day 1, patients self‐administered chlormethine gel 0.016% (EMA‐approved) daily for 16 consecutive weeks. Patients returned for study visits at Weeks 4, 8, 12 and 16. A comprehensive list of inclusion and exclusion criteria for both studies is shown in Tables S1 and S2.

### 2.3 Clinical Scoring

Disease severity was assessed using the mSWAT and CAILS score. In the confirmation cohort, response to topical chlormethine was evaluated by change from baseline on the modified CAILS (mCAILS). The mCAILS was used to monitor the primary lesion, as it excludes the individual score for hypo‐ or hyperpigmentation to avoid misclassifying post‐inflammatory hyperpigmentation as active disease. Clinical response was defined by standard oncology criteria as over 50% lesion improvement on mCAILS compared to baseline [28]. Clinical scoring was performed at all time points by one of two trained investigators, each blinded to previous scores. Two dermatologists specialised in CTCL (MV and KQ) performed training and supervision.

### 2.4 Imaging Lesion Characterisation

Target lesions and contralateral non‐lesional skin were characterised using non‐invasive imaging under standardised conditions. Devices were calibrated following manufacturer guidelines. Clinical photographs were taken at all time points with an EOS700 SLR camera (Canon, United Kingdom). Multispectral imaging (MSI, Antera 3D, Miravex Limited, Ireland) quantified skin erythema by CIELAB a* and average haemoglobin, skin pigmentation by average melanin, plaque elevation by maximum elevation and desquamation by average roughness. MSI uses the CIELAB colour classification system, where the a* value (AU) indicates erythema and the *m value (AU) represents melanin [29]. A formula derived from the L* and b* values was used to calculate the individual typology angle (ITA) [29, 30]. A 5 × 5cm^2^ closed chamber captured images of each target lesion over time with standardised distance and lighting [31, 32], maintaining a consistent 30 mm^2^ region of interest (ROI). In total, 173 lesional and 165 non‐lesional images were captured. Colourimetry (DSM III ColourMeter, Cortex Technology, Denmark) was used in the confirmation cohort to measure erythema using an RGB sensor over 7 mm^2^ skin surface [33]. The mean of triplicate measurements was calculated on all time points at a standardised part of the lesion. Laser speckle contrast imaging (LSCI, PeriCam, PSI NR system, Perimed AB, Sweden) was added to the confirmation cohort to assess microvascular perfusion up to 700 μm depth [34, 35, 36, 37]. Patients acclimatised in a temperature‐controlled room for 15 min, followed by 30 s of LSCI recordings taken at a consistent distance from the lesion ROI at each time point.

### 2.5 Patient Acceptability

A validated burden questionnaire was used to assess patient acceptability at the end of the confirmation cohort. The experienced burden of each device was rated on a visual analogue scale ranging from 0 (no burden) to 100 (maximum burden) (Figure S3) [38, 39]. Candidate imaging biomarkers were considered acceptable if the burden score was 20 or lower [23].

### 2.6 Statistics

The discovery cohort listed all variables by subject, group (MF patch, MF plaque, MF tumour and healthy controls) and location (lesional and non‐lesional). Continuous variables were analysed with a one‐way analysis of variance (ANOVA) with group as factor. All calculations were performed using SAS V9.4 for Windows (SAS Institute Inc., Cary, NC, USA) and R (R Foundation for Statistical Computing, Vienna, Austria).

In the confirmation cohort, all candidate imaging biomarkers were summarised by treatment group (lesional skin and non‐lesional skin within MF patients, or lesional skin within MF patients and healthy controls or non‐lesional skin of MF patients and healthy controls) and protocol time using descriptive statistics. Data from patients who withdrew were included in the responder analyses using the last‐observation‐carried‐forward method.

The inferential analysis in the observational study part was conducted by fitting a mixed model analysis of variance with treatment group, protocol time and interaction of treatment group by protocol time as fixed factors and random factor subject, and for comparison within MF patients subject by time and subject by group. Variance‐component type of variance–covariance structure was used. The estimated variances were used to calculate (i) coefficient of variation (CV) expressed as inter‐ or intra‐subjects variability divided by corresponding least square mean and intra‐class correlation (ICC) expressed as intra‐subjects variabilities divided by total variability (inter‐subjects variability plus intra‐subjects variabilities), and (ii) the minimal detectable effect size (MDES) assuming a power of 80%, and alpha of 0.05 two‐sided and 20 subjects.

Repeated measures correlation (*rmcorr* package in R) was used to assess the within‐subject association of paired measures evaluated at multiple timepoints. *Rmcorr* accounts for the non‐independence of repeated measures using analysis of covariance (ANCOVA) to statistically correct for inter‐subject variability. By removing measured variance between‐subjects, *rmcorr* ensures the best linear fit for each subject using parallel regression lines (same slope) with varying intercepts. The repeated measures correlation coefficient was based on the estimated sum of squares for both the measures and error [40].

### 2.7 Criteria for Biomarkers

**FIGURE 1:**
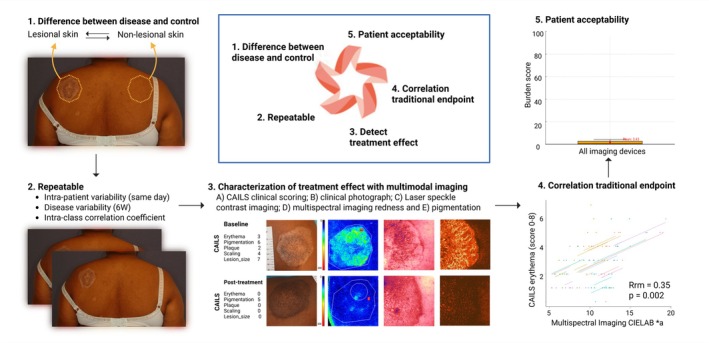
Biomarker validation overview. Clinical validation criteria for candidate imaging biomarkers in cutaneous T‐cell lymphomas. Criteria following the Kruizinga methodology were used to analyse the confirmation cohort dataset: (1) Statistically significant difference between diseased and non‐diseased control skin; (2) repeatable, defined as a coefficient of variation (CV) ≤ 10% within the MF lesion group and intra‐class correlation (ICC) > 75%; (3) sensitive to changes in disease activity and treatment response; (4) correlate with traditional endpoints, critically appraising data and accounting for limitations of the novel candidate biomarkers and/or the ‘gold standard’; (5) patient acceptability, with burden scores for the undergoing tests ≤ 20 on a 100‐point scale.

Imaging tests were validated as biomarkers when fulfilling the following requirements outlined in the Kruizinga methodology [23] (Figure 1): (i) a statistically significant difference between diseased and non‐diseased control skin; (ii) repeatability, defined as a coefficient of variation (CV) ≤ 10% within the MF lesion group and ICC > 75%; (iii) sensitivity to changes in disease activity and treatment response; (iv) correlation with traditional endpoints, critically appraising data and accounting for limitations of the novel candidate biomarkers and/or the ‘gold standard’; (v) patient acceptability, with burden scores for the undergoing tests ≤ 20 on a 100‐point scale.

## 3 Results

### 3.1 Baseline Characteristics

A total of 56 MF patients and 10 healthy volunteers participated in both studies. The discovery cohort included 16 patients with patches, 12 with plaques and 7 with non‐ulcerative tumours, with no dropouts. The confirmation cohort comprised 21 early‐stage MF patients, of whom 19 completed the full 16 week follow‐up. Two patients withdrew earlier due to recurrent application site reactions, resulting in a mean follow‐up time of 15.4 weeks. Patient characteristics, including a mean age of 58.91 years (range 19–87) and Fitzpatrick skin types I–V, are displayed in Table 1.

**TABLE 1:** Demographics and baseline characteristics.

Characteristics	Discovery cohort (*n* = 35)	Confirmation cohort (*n* = 21)	Both cohorts (*N* = 56)	Healthy controls (*N* = 10)
Age, years				
Mean (SD, range)	60.4 (15.0, 24–84)	52.9 (14.3, 19–72)	58.91 (15.5, 19–87)	54.2 (18.2, 22–73)
Sex, *N* (%)				
Female	11 (31.4)	9 (42.9)	25 (39.1)	5 (50.0)
Male	24 (68.6)	12 (57.1)	39 (60.9)	5 (50.0)
Fitzpatrick skin type, *N* (%)				
I	5 (14.3)	2 (9.5)	8 (12.5)	0
II	24 (68.6)	12 (57.1)	43 (67.2)	4 (40.0)
III	2 (5.7)	4 (19.0)	6 (9.4)	6 (60.0)
IV	3 (8.6)	1 (4.8)	4 (6.3)	0
V	1 (2.9)	2 (9.5)	3 (4.7)	0
VI	0	0	0	0
Target location, *N* (%)				
Head	3 (8.6)	0	3 (4.7)	0 (0.0)
Arm	2 (5.7)	0	3 (4.7)	0 (0.0)
Trunk	12 (34.3)	7 (33.4)	21 (32.8)	4 (40.0)
Swimsuit area	10 (28.6)	4 (19.0)	16 (25)	3 (30.0)
Upper leg	7 (20.0)	6 (28.6)	15 (23.4)	2 (20.0)
Lower leg	1 (2.9)	4 (19.0)	6 (9.4)	1 (10.0)
MF stage, *N* (%)				
IA	5 (14.3)	13 (61.9)	18 (32.1)	NA
IB	10 (28.6)	8 (38.1)	18 (32.1)	
IIA	0	0	0	
IIB	17 (48.6)	0	17 (30.4)	
IVA1	3 (8.6)	0	3 (8.6)	
IVA2	0	0	0	
IVB	0	0	0	
Target lesion, *N* (%)				
Patch	16 (37.2)	18 (85.7)	34 (53.1)	NA
Plaque	12 (27.9)	3 (14.3)	15 (23.4)	
Tumour	7 (16.3)	0	7 (10.9)	
CAILS, mean (SD, range)				
Patch	14.1 (4.0, 7–23)	15.9 (3.4, 9–22)	15.0 (3.8, 7–23)	NA
Plaque	14.0 (5.5, 5–27)	18.7 (2.6, 14–22)	14.9 (5.5, 5–27)	
Tumour	17.3 (3.6, 12–22)	0	17.4 (3.7, 12–22)	
mCAILS, mean (SD, range)				
Patch	12.6 (3.6, 7–21)	14.5 (3.3, 8.0–20.0)	13.6 (3.6, 7–21)	NA
Plaque	12.8 (5.5, 5–26)	16.3 (3.5, 13–20)	13.5 (5.3, 5–26)	
Tumour	17.0 (3.6, 12–22)	0	17.0 (3.6, 12–22)	
mSWAT, mean (SD, range)				
Patch	11.9 (15.8, 2–67)	13.6 (17.1, 2–79)	12.8 (16.3, 2–79)	NA
Plaque	14 (16.8, 2–60)	7.67 (3.8, 5–12)	12.7 (15.2, 2–60)	
Tumour	20.9 (17.0, 6–56)	0	20.9 (17.0, 6–56)	

*Note:* Demographic data and baseline characteristics of the study population, including age, sex, Fitzpatrick scale, target lesion and clinical scores. Data are expressed as mean (standard deviation) or frequency (percentage), as appropriate.

Abbreviations: CAILS, composite assessment of index lesion severity; CTCL, cutaneous T‐cell lymphoma; mCAILS, modified composite assessment of index lesion severity; MF, mycosis fungoides; mSWAT, modified severity‐weighted assessment tools.

### 3.2 Discrimination of Lesional and Non‐Lesional Skin

**FIGURE 2:**
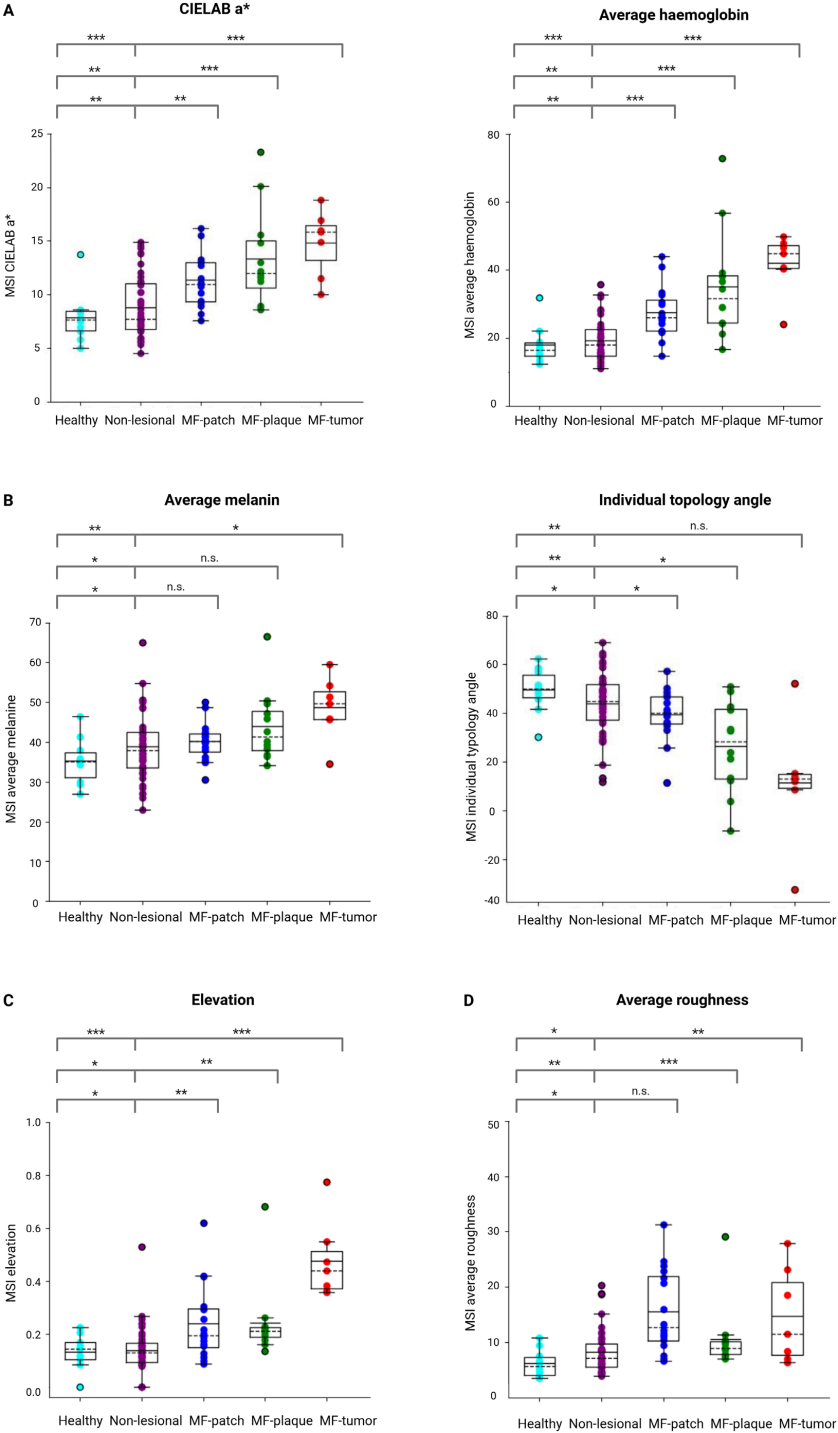
Quantification of Composite Assessment of Index Lesion Severity parameters using multispectral imaging. Multispectral imaging (MSI) was used in the discovery cohort to objectively quantify Composite Assessment of Index Lesion Severity (CAILS) parameters by analysing the following candidate biomarkers: (A) Erythema, quantified by CIELAB a* and average haemoglobin. (B) pigmentation, quantified by average melanin and Individual Typology Angle (ITA). (C) elevation, quantified by maximum elevation. (D) scaling, quantified by average roughness. The boxplots show comparisons between healthy control skin, non‐lesional skin, and lesional skin, including MF‐patch, MF‐plaque, MF‐tumour. Statistical significance is indicated as follows: ns = not significant, *p < 0.05, **p < 0.01, ***p < 0.001.

In the discovery cohort, all candidate imaging biomarkers effectively distinguished erythema, elevation, pigmentation and scaling, comparing lesional skin to contra‐lateral non‐lesional skin and healthy controls (Figure 2 and Table S3). Further evaluation in the confirmation cohort revealed increased erythema in lesional skin compared to non‐lesional skin: MSI CIELAB a* (Δ2.39, 1.08–3.70, *p* = 0.0011), average haemoglobin (Δ7.86, 4.72–11.01, *p* < 0.0001), colourimetry CIELAB a* (Δ5.41, 3.48–7.34, *p* < 0.0001), and LSCI microcirculation (Δ8.43, 2.64–14.23, *p* = 0.0065) (Table 2). MSI average melanin showed no significant difference in pigmentation (Δ2.23, −1.22 to 5.69, *p* = 0.1924). ITA effectively discriminated lesional from non‐lesional skin (Δ‐6.10, −11.50 to −0.80, *p* = 0.0259). MSI maximum elevation and average roughness failed to significantly differentiate lesional from non‐lesional skin (Δ0.06, −0.01 to 0.13, *p* = 0.0872 and Δ2.25, −0.33 to 4.82, *p* = 0.0837, respectively).

**TABLE 2:** Systematic evaluation of skin assessment methods for biomarker suitability.

Candidate digital biomarker	1. Discrimination disease and control				2. Repeatability			3. Detect treatment effect	4. Correlation to traditional endpoint			Biomarker
	Lesion mean	NL mean	*p*	95% CI	Intra‐patient CV (intra‐day)	Intra‐patient CV (6 W)	ICC	MDES	Rrm	*p*	95% CI	
*Erythema*												
MSI CIELAB a*	10.86	8.47	0.0011	1.08; 3.70	7.4%	8.1%	0.89	2.58	0.35	0.002	0.14; 0.53	Yes
MSI average haemoglobin	26.70	18.84	< 0.0001	4.72; 11.01	7.5%	8.9%	0.88	7.33	0.37	0.0002	0.19; 0.53	Yes
Colourimetry CIELAB a*	18.70	13.30	< 0.0001	3.48; 7.34	6.4%	7.0%	0.84	3.75	0.32	0.004	0.11; 0.50	Yes
LSCI microcirculation (AU)	52.57	44.14	0.0065	2.64; 14.23	18.2%	10.6%	0.73	15.32	0.40	0.0002	0.20; 0.57	Inconclusive
*Pigmentation*												
MSI average melanin	40.36	38.13	0.1924	−1.22; 5.69	2.2%	4.4%	0.98	11.70	0.04	0.70	−0.16; 0.23	No
MSI ITA (degrees)	38.30	44.40	0.0259	−11.5; −0.80	9.5%	7.7%	0.98	23.03	0.02	0.83	−0.18; 0.22	Yes
*Elevation*												
MSI maximum elevation	0.18	0.12	0.0872	−0.01; 0.13	20.2%	11.7%	0.91	0.14	0.11	0.32	−0.11; 0.32	Inconclusive
*Desquamation*												
MSI average roughness	9.59	7.35	0.0837	−0.33; 4.82	13.0%	17.9%	0.78	5.88	0.30	0.008	0.08; 0.48	Inconclusive

*Note:* Systematic overview of various skin assessment methods used in the confirmation cohort, evaluating their potential as biomarkers based on Kruizinga's methodology. The evaluation considers discrimination between disease and control, repeatability, treatment effect detection, and correlation with existing traditional clinical endpoints. Colour coding: Green indicates suitability; red indicates unsuitability; yellow indicates an indeterminate outcome.

Abbreviations: AU, arbitrary units; CI, confidence interval; CV, coefficient of variation; ICC, intra‐class correlation coefficient; ITA, individual typology angle; LSCI, laser speckle contrast imaging; MDES, minimal detectable effect size; MSI, multispectral imaging; NL, non‐lesional skin; Rrm, repeated measures correlation.

### 3.3 Repeatability

Most candidate biomarkers demonstrated low intra‐patient variability (CV < 10%) and good (ICC 0.75–0.90) to excellent (ICC ≥ 0.90) reliability. Repeatability of LSCI microcirculation was more variable with 18.2% intra‐day variance, 10.6% variability over 6 weeks, and a moderate ICC of 0.73. MSI average roughness and maximum elevation showed high variability in intra‐patient CVs but displayed good (ICC 0.78) to excellent (ICC 0.91) reliability.

### 3.4 Responsive to Treatment Effect

**FIGURE 3:**
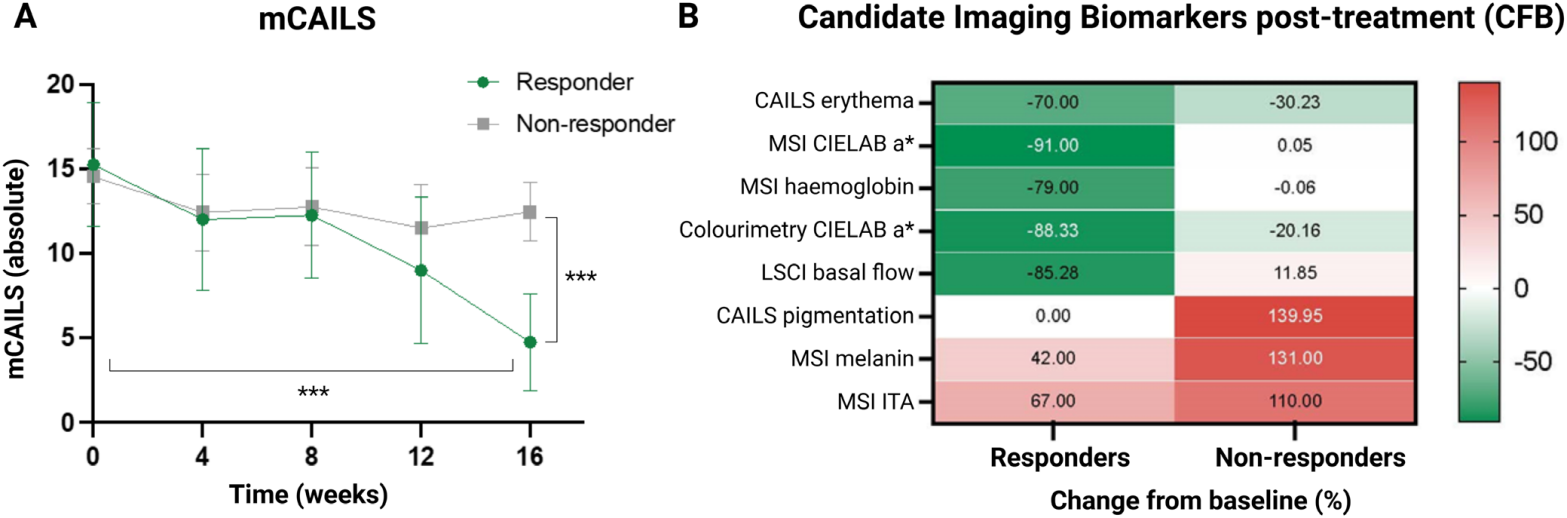
Treatment effects evaluated by clinical scoring (mCAILS) and by candidate imaging biomarkers. Treatment response to chlormethine gel was evaluated in 21 patients with mycosis fungoides using the modified Composite Assessment of Index Lesion Severity (mCAILS) score and candidate imaging biomarkers. (A) Using mCAILS, a significant treatment effect was observed in eight responders (Δ‐10.5 ± 3.412, *p* < 0.0001) over 16 weeks. Comparing clinical scores at Week 16 between eight responders and 13 non‐responders, responders showed a significantly greater reduction in mCAILS scores (Δ‐2.08 ± 2.876, *p* < 0.0001). (B) The percentage change from baseline (CFB) in candidate imaging biomarkers at the end of the study is illustrated in this heatmap, stratified by clinical response status (responders vs. non‐responders). Candidate biomarkers include MSI CIELAB a*, haemoglobin, melanin, Individual Typology Angle (ITA), colourimetry CIELAB a* and LSCI microcirculation. Lesional parameters were corrected for the corresponding non‐lesional values of each patient. A 100% decrease reflects normalisation to levels comparable to healthy control skin within the same individual.

Physician‐reported efficacy, reflected by a significant decrease in mCAILS, was observed in 8 patients after 16 weeks of treatment (−10.5 ± 3.4, *p* < 0.0001), significantly differing from the 13 non‐responders (−2.1 ± 2.8, *p* < 0.0001) (Figure 3). As outlined in the heatmap (Figure 3B), candidate imaging biomarkers detected clear treatment effects when comparing change from baseline of the responder to the non‐responder group. Clinical scoring of CAILS erythema and the decrease of more than 78% in erythema for all candidate biomarkers in the responder group indicate adequate detection of disease remission.

**FIGURE 4:**
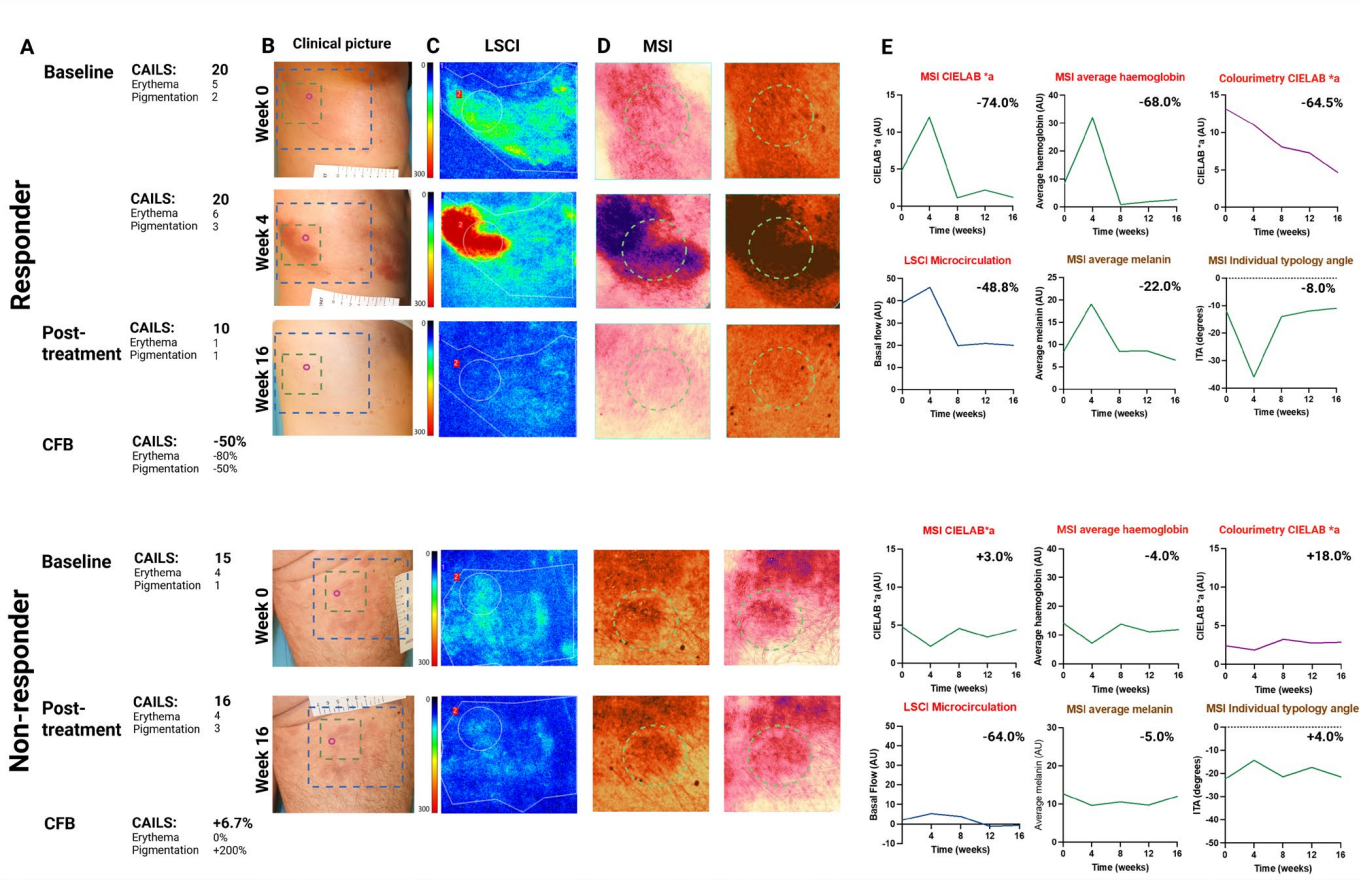
Treatment effects in all candidate imaging biomarkers in a responder and non‐responder. (A) Lesional severity scoring over time by CAILS, illustrating change from baseline (CFB). (B) Clinical photographs with regions of interest (ROIs) highlighted: blue dotted areas indicate the ROI for laser speckle contrast imaging (LSCI), green dotted areas for multispectral imaging (MSI) and the purple circle for colourimetry. (C) LSCI images visualising lesional microvasculature. (D) MSI erythema and pigmentation images of a partial lesion. The green dotted circle marks the ROI used for digital biomarker quantification. (E) Candidate biomarkers associated with erythema are titled in red and demonstrate a clear peak at Week 4, followed by decreased erythema post‐treatment in the responder. Colours of the lines correspond to their respective ROIs in images (B and D). Candidate biomarkers quantifying pigmentation are titled in brown. ITA is inverse to quantified pigmentation. Candidate biomarkers are corrected for the mean non‐lesional value of the respective patient, meaning a 100% decrease indicated normalisation of these parameters to the level observed in healthy control skin of the respective individual.

A responder was selected that exhibited an initial increase in lesional erythema and pigmentation, followed by a decrease due to lesion improvement (Figure 4). All candidate biomarkers quantifying erythema and pigmentation detected these changes, reaffirming their responsiveness to disease activity. In contrast, a non‐responsive patient showed stable biomarker measurements over time. MDESs between lesional and non‐lesional skin were calculated for all biomarkers (Table 2). The most sensitive candidate biomarker was colourimetry CIELAB a*, since the calculated MDES as a proportion of the respective group difference was the smallest.

### 3.5 Correlation to CAILS as Traditional Endpoint

Most candidate imaging biomarkers showed positive within‐subject correlations with the corresponding subscores of the traditional CAILS endpoint; however, of poor to fair strength (Table 2). Fair within‐subject correlations for erythema severity were detected with MSI CIELAB a* (Rrm = 0.35, *p* = 0.002, 95% CIs = [0.14; 0.53]), average haemoglobin (Rrm = 0.37, *p* = 0.0002, 95% CIs = [0.19; 0.53]), colourimetry CIELAB a* (Rrm = 0.32, *p* = 0.004, 95% CIs = [0.11; 0.50]), and LSCI microcirculation (Rrm = 0.40, *p* = 0.0002, 95% CIs = [0.20; 0.57]). Pigmentation‐related tools did not correlate with clinical scoring of CAILS pigmentation. Candidate biomarkers quantifying erythema and pigmentation correlated within‐subjects fairly to strongly with each other (Figure S4). Descriptive analysis of MSI parameters across grouped Fitzpatrick skin types (I + II, III, IV + V) showed that CIELAB a* and average haemoglobin remained relatively consistent across groups, while average melanin appropriately increased with darker skin types (Figure S5). Clinically scored plaque elevation was not correlated within‐subject in MSI maximum elevation (Rrm = 0.11, *p* = 0.32, 95% CIs = [−0.11; 0.32]). Lastly, desquamation scoring within‐subjects was fairly correlated to MSI average roughness (Rrm = 0.30, *p* = 0.008, 95% CIs = [0.08; 0.48]).

### 3.6 Patient Acceptability

The experienced burden by MF patients from the non‐invasive measurements was low for all devices. Clinical photographs and multispectral imaging had a mean burden score of 4.1 out of 100 (IQR 5.0) and LSCI had a mean score of 2.7 (IQR 3.0).

## 4 Discussion

This study successfully identified candidate imaging biomarkers in 35 MF patients and ultimately validated those for assessing erythema and pigmentation in 21 early‐stage MF patients. CIELAB a* and average haemoglobin of MSI, as well as CIELAB a* by colourimetry, met all predefined clinical validation criteria for quantifying erythema in MF. Especially the colourimeter, a compact handheld device providing rapid results, is highly suitable as a practical imaging biomarker in clinical settings. LSCI microcirculation demonstrated clear group differences and a significant correlation to CAILS erythema, confirming its potential as an objective measure of vascular changes in MF. However, slightly increased within‐lesion variability was observed, warranting further validation before it can be considered a stand‐alone biomarker for MF. No biomarkers were identified to objectively quantify lesion elevation and desquamation. ITA by MSI was validated for quantifying pigmentation.

Although the erythema biomarkers offer objective and reproducible alternatives to clinical scoring, the reliability of pigmentation as a disease severity parameter in classical MF remains uncertain. Lesions can present with both hypo‐ and hyperpigmentation, making consistent scoring within the same CAILS subscore challenging. Moreover, hyperpigmentation may indicate lesion progression, as observed in 58.6% of CD8+ cytotoxic MF cases [41], or reflect post‐inflammatory changes after treatment with topical chlormethine [42, 43, 44]. The lack of correlation between imaging biomarkers and the CAILS pigmentation subscore underscores its limited reliability, reinforcing previous recommendations to exclude pigmentation as a clinical parameter from CAILS scoring [28].

A key advantage of imaging biomarkers over traditional clinical endpoints is their excellent repeatability, contrasting sharply with the high intra‐ and inter‐rater variability commonly seen in clinical scoring methods like CAILS [7, 8, 9, 10, 45]. Bożek et al. examined inter‐rater variability in atopic dermatitis severity scoring by 10 trained dermatologists at two time points, finding moderate variability for IGA (CV 33.0%) and oSCORAD (CV 28.1%), and high variability for EASI (CV 66.5%) [7]. Similar high variability likely applies to CAILS, raising concerns about its reliability as a primary outcome measure. Moreover, dermatological clinical scoring in open‐label study designs is constantly prone to response quantification bias, which may have weakened the repeated measures correlations between objective device metrics and subjective CAILS subscores. Additionally, it is important to acknowledge that perfect correlations are unlikely due to the inherent differences between traditional endpoints and imaging biomarkers [22]. Nonetheless, these devices have undergone thorough technical validation and have been extensively tested in previous clinical trials across various diseases [29, 30, 31, 32, 34, 35, 36, 46, 47, 48, 49, 50, 51, 52, 53, 54]. This supports their ability to objectively quantify disease measures, whereas the reliability of CAILS scores as a traditional endpoint remains debatable.

Several limitations must be considered. First, the confirmation cohort consisted exclusively of early‐stage MF patients, primarily with patch‐type lesions due to the study design incorporating chlormethine gel, a therapy mainly prescribed in very early stages. The absence of identified biomarkers for lesion elevation and desquamation is likely due to the flat morphology and minimal scaling characteristic of these lesions [28]. As elevation and desquamation become more pronounced in later disease stages, future studies should include patients with more advanced disease to validate biomarkers for these features. Second, our study predominantly included patients with a lighter skin phototype, making the applicability to darker skin types uncertain. While our descriptive analysis (Figure S5) suggests that MSI derived erythema parameters remain relatively independent of constitutive pigmentation across grouped Fitzpatrick skin types, this finding should be interpreted cautiously given the imbalanced skin type distribution in our cohort. The technical independence of melanin and haemoglobin measurements is supported by the spectral separation capabilities of the MSI across multiple wavelengths [55]. However, prospective validation in adequately powered studies with stratified enrolment across all Fitzpatrick categories remains necessary to definitively establish the performance of these biomarkers across diverse skin types. Third, novel imaging biomarkers, such as MSI and colourimetry, are limited by the fixed measurement areas. Due to the morphological heterogeneity of MF lesions, these areas might not represent the region most reflective of erythema as scored by CAILS, as illustrated in Figure 4B,E. For example, colourimetry missed the erythema peak in a responder at Week 4 due to its small and inconsistent size. Fourth, varying anatomical target sites cause inter‐patient variability. Especially LSCI microcirculation showed increased variability, in contrast with previous studies that reported low variability and high discriminating power for LSCI measurements [34, 46, 47]. These findings may be attributed to differences in measurement protocols, such as an extended measurement duration causing motion artefacts. Optimised protocols may improve reliability. Finally, evaluation of clinical response at 16 weeks may be suboptimal, as CAILS by‐time analyses from the chlormethine gel pivotal study showed a steady increase in response rates each month, peaking at 10 months [54]. A longer follow‐up period may be necessary to accurately assess biomarker performance. Despite these limitations, this study highlights the potential of imaging biomarkers as objective and reliable tools for monitoring MF and treatment effect. Future studies should include patients with advanced stage MF, diverse skin phototypes, and longer follow‐up duration (NCT07091604 and NCT07021495). Integrating biomarkers with established prognostic indices, such as PROCLIPI [56] and S‐MISR [57], could further confirm their long‐term clinical relevance.

## 5 Conclusion

This study presents a structured approach to validating novel non‐invasive imaging biomarkers for MF. Based on our findings, MSI CIELAB a*, MSI average haemoglobin and colourimetry CIELAB a* are recommended for the objective quantification of erythema, and MSI ITA for pigmentation. These biomarkers provide a more accurate and sensitive quantification of disease extent in MF compared to conventional clinical scoring systems. Given the heterogeneous phenotype of CTCL, incorporating these biomarkers as endpoints in future clinical trials is recommended to enhance the reliability of outcome measures.

## Author Contributions

**Selinde S. Wind:** study setup, study execution, data analysis, manuscript writing, manuscript review and approval; **Elise S. M. Beljaards:** data analysis, manuscript writing, manuscript review and approval; **Rianne Rijneveld:** study setup, study execution, data analysis, manuscript review and approval; **Lisa Bruijnincx:** study setup, study execution, data analysis, manuscript review and approval; **Tessa Niemeyer‐van der Kolk:** study setup, study execution, data analysis, manuscript review and approval; **Manon A. A. Jansen:** study setup, study execution, data analysis, manuscript review and approval; **Yalcin Yavuz:** study setup, data analysis, manuscript review and approval; **Marieke de Kam:** study setup, data analysis, manuscript review and approval; **Jacobus Burggraaf:** supervision of all study activities, manuscript review and approval; **Naomi Klarenbeek:** medical responsibility, manuscript review and approval; **Jacobus Bosch:** medical responsibility, manuscript review and approval; **Koen D. Quint:** supervision, study setup, study execution, data analysis, manuscript writing, manuscript review and approval; **Maarten H. Vermeer:** supervision, study setup, study execution, data analysis, manuscript writing, manuscript review and approval; **Robert Rissmann:** principal investigator of the clinical studies, study setup, study execution, data analysis, manuscript writing, manuscript review and approval.

## Funding

This project was funded by CHDR R&D fund and supported by Recordati Rare Diseases/Helsinn and Micreos Pharma BV. This study is part of the SKINERGY CTCL study and partially funded by the Dutch Research Council (NWO), NWA‐ORC programme with project NWA.1389.20.182 entitled Next Generation ImmunoDermatology (NGID).

## Ethics Statement

The studies were approved by the independent Dutch Medical Ethics committee and all patients provided written informed consent prior to any study‐related activity. Clinicaltrial.gov identifiers: NCT05303480 and NCT05827107.

## Conflicts of Interest

The authors declare no conflicts of interest.

## Supporting information


**Figure S1:** Structured approach to validate candidate digital imaging biomarkers [25, 26, 27, 28]. A structured approach was employed to validate reliable biomarkers for clinical trial applications. Candidate biomarkers were identified based on baseline characteristics from a discovery cohort consisting of 35 patients with mycosis fungoides (MF) and 10 healthy volunteers. Using multispectral imaging, potent biomarkers were established for quantifying the parameters of the Composite Assessment of Index Lesion Severity (CAILS), including erythema, pigmentation, elevation and scaling. In a confirmation cohort of 21 early‐stage MF patients treated with chlormethine gel, candidate biomarkers for quantifying erythema and pigmentation were further validated using multispectral imaging and colorimetry. Finally, a critical appraisal was conducted to compare these novel biomarkers with traditional clinical endpoints.

**Figure S2:** Clinical validation of imaging and traditional biomarkers. Stepwise validation process of imaging‐based and traditional biomarkers. The framework includes potential biomarker selection, technical validation, clinical validation and evaluation as a candidate biomarker in clinical trials.

**Figure S3:** Burden questionnaire for assessing patient acceptability. Burden questionnaire used to evaluate patient acceptability of various skin assessment methods. The questionnaire includes subjective burden ratings for invasive and non‐invasive skin measurements, as well as procedures conducted at home. Patients were asked to indicate their perceived burden on a visual analogue scale ranging from ‘not burdensome at all’ to ‘most burdensome ever’.

**Figure S4:** Correlation of candidate biomarkers for quantifying erythema and pigmentation. The repeated measures correlation between candidate parameters for quantifying erythema (CIELAB a*, average haemoglobin, microcirculation) and pigmentation (average melanin and Individual Typology Angle [ITA]) using multispectral imaging (MSI), colourimetry, and laser speckle contrast imaging (LSCI) is depicted in these repeated measures correlation plots. Each dot represents an individual data point, with different colours indicating different subjects. The lines represent individual regression fits within each subject. (A) MSI average haemoglobin shows a strong positive within‐subject correlation with MSI CIELAB a* (Rrm = 0.95, 95% CIs = [0.93; 0.97], *p* < 0.0001). (B) A weaker within‐subject correlation is observed between MSI average haemoglobin and colourimetry CIELAB a* (Rrm = 0.29, 95% CIs = [0.10; 0.46], *p* = 0.0029). (C) A moderate within‐subject correlation is found between MSI average haemoglobin and LSCI microcirculation (Rrm = 0.50, 95% CIs = [0.34; 0.64], *p* < 0.0001). (D) A strong negative within‐subject correlation is observed between MSI Individual Typology Angle (ITA) and MSI average melanin (Rrm = −0.87, 95% CIs = [−0.91; −0.81], *p* < 0.0001).

**Figure S5:** Skin colour related parameters measured by multispectral imaging, stratified by Fitzpatrick skin type. (A) Mean CIELAB a⁎ values, (B) mean average haemoglobin levels, and (C) mean average melanin levels measured within a 30.0 mm region of interest (ROI) using multispectral imaging. Data are shown for Fitzpatrick skin types I–II, III, and IV–V. Black dots represent group means, with error bars indicating variability within each group (mean ± SD). An increasing trend is observed across Fitzpatrick skin types, most prominently for average melanin.

**Table S1:** Inclusion criteria. Specific criteria required for study participation.

**Table S2:** Exclusion criteria. Criteria that disqualify individuals from participating in the study.

**Table S3:** Outcomes of multispectral imaging as a candidate imaging biomarker to quantify CAILS parameters. Outcomes of multispectral imaging as a potential imaging biomarker for assessing Composite Assessment of Index Lesion Severity (CAILS) parameters. It presents the differences (Δ) and confidence intervals (CI) for comparisons between lesional skin and healthy controls, as well as lesional and non‐lesional skin. Statistical significance is indicated by *p*‐values, with significant results highlighted. Colour coding: Green indicates statistically significant results; red indicates non‐significant results. Abbreviations: CI, confidence intervals; CAILS, Composite Assessment of Index Lesion Severity; MSI, multispectral imaging.

## Data Availability

The data supporting the findings of this study are available on reasonable request at the author in accordance with local privacy laws (GDPR).

## Appendix A

Collaborators (PubMed Indexation): Rob Vreeken, Maastricht University; Eva Cuypers, Maastricht University; Sylvia van Beugen, Leiden University; Antoinette van Laarhoven, Leiden University; Boudewijn Lelieveldt, Leiden University Medical Center; Abdoel El Ghalbzouri, Leiden University Medical Center; Marieke Seyger, Radboud University Medical Center; Juul van den Reek, Radboud University Medical Center; Ellen van den Bogaard, Radboud University Medical Center; Elke de Jong, Radboud University Medical Center; Peter van den Broek, HuidNederland; Bernd Arents, VCME; Ton de Leeuw, Stichting Huidlymfoom; Tom Ederveen, Radboud UMC; Peter Bram 't Hoen, Radboud UMC; Annemarie Sluijmers, Nationale vereniging voor mensen met lupus, APS, sclerodermie en MCTD; Bernd Arents, Vereniging voor Mensen met Constitutioneel Eczeem; Mieke de Leeuw, Stichting Huidlymfoom; Ton de Leeuw, Stichting Huidlymfoom; Peter van den Broek, Huid Nederland en Patiëntenplatform Urticaria; Els van der Pool, Hogeschool Arnhem‐Nijmegen; Ellen Stijl‐'t Hart, Leiden University Medical Center; Lisanne Wezendonk, Leiden University Medical Center; Douwe Vellinga, Alrijne Hospital Leiderdorp; Stephan Weidinger, University Hospital Kiel Schleswig Holstein; Sandrine Dubrak, Medical University of Innsbruck; Yang Li, Helmholtz Centre for Infection Research; Maurice Steensel, Nanyang Technological University, Singapore; Martina Marchetti‐Deschmann, Technical University Wien.
