# Supplementary figures and images for: Clinical Validation of Imaging Biomarkers in Mycosis Fungoides
Source: Exp Dermatol. 2026 Mar 11;35(3):e70236. doi: 10.1111/exd.70236 (PMC12977146; doi:10.1111/exd.70236)

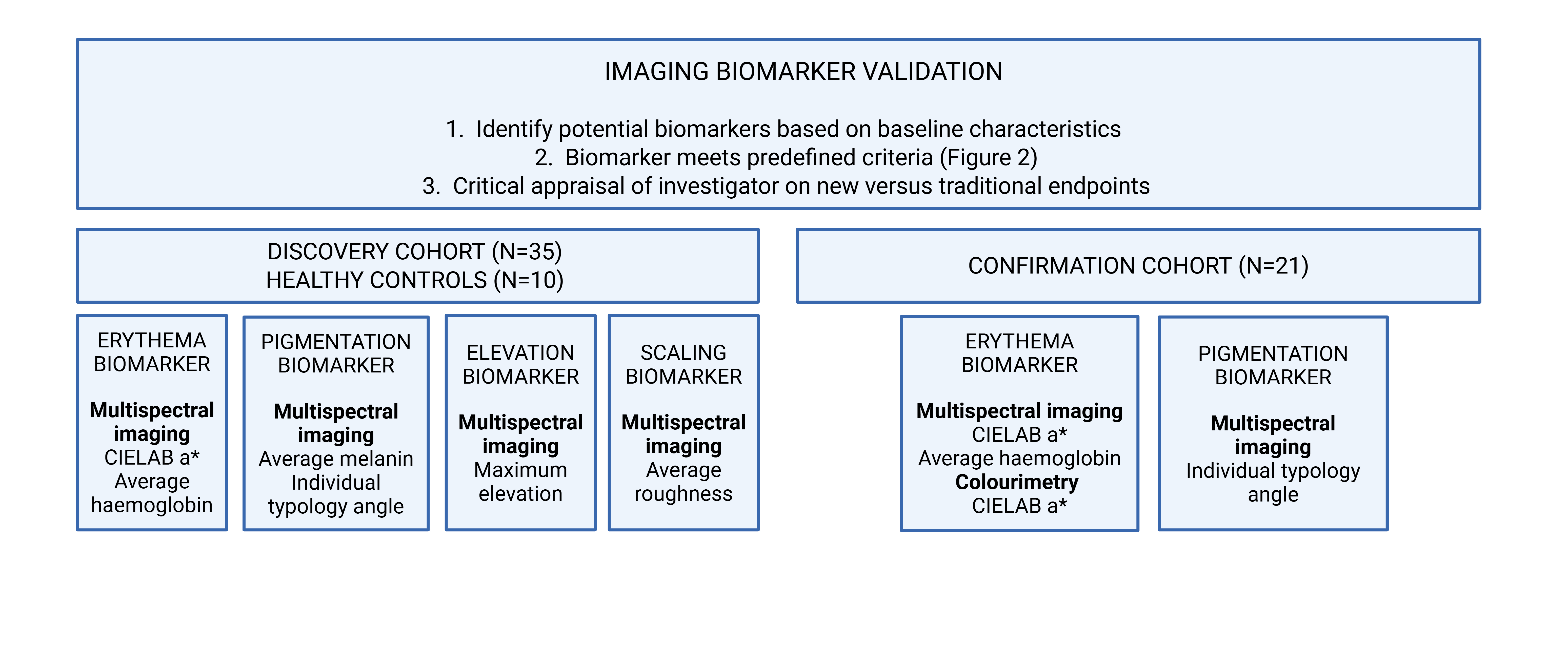

Supplement: Supplementary file 1 — Figure S1: Structured approach to validate candidate digital imaging biomarkers [25, 26, 27, 28]. A structured approach was employed to validate reliable biomarkers for clinical trial applications. Candidate biomarkers were identified based on baseline characteristics from a discovery cohort consisting of 35 patients with mycosis fungoides (MF) and 10 healthy volunteers. Using multispectral imaging, potent biomarkers were established for quantifying the parameters of the Composite Assessment of Index Lesion Severity (CAILS), including erythema, pigmentation, elevation and scaling. In a confirmation cohort of 21 early‐stage MF patients treated with chlormethine gel, candidate biomarkers for quantifying erythema and pigmentation were further validated using multispectral imaging and colorimetry. Finally, a critical appraisal was conducted to compare these novel biomarkers with traditional clinical endpoints. [file EXD-35-e70236-s008.jpeg]

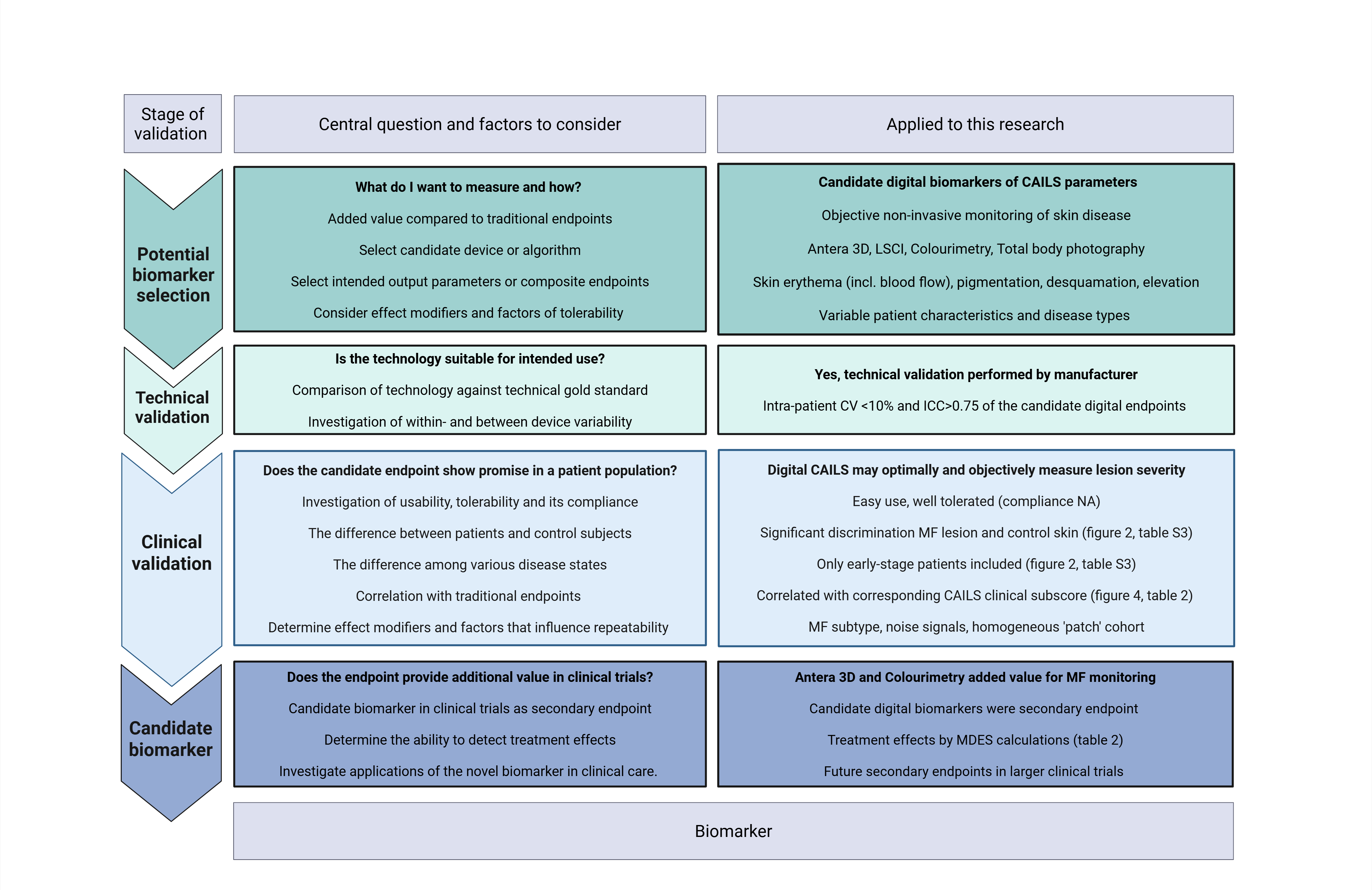

Supplement: Supplementary file 2 — Figure S2: Clinical validation of imaging and traditional biomarkers. Stepwise validation process of imaging‐based and traditional biomarkers. The framework includes potential biomarker selection, technical validation, clinical validation and evaluation as a candidate biomarker in clinical trials. [file EXD-35-e70236-s003.jpeg]

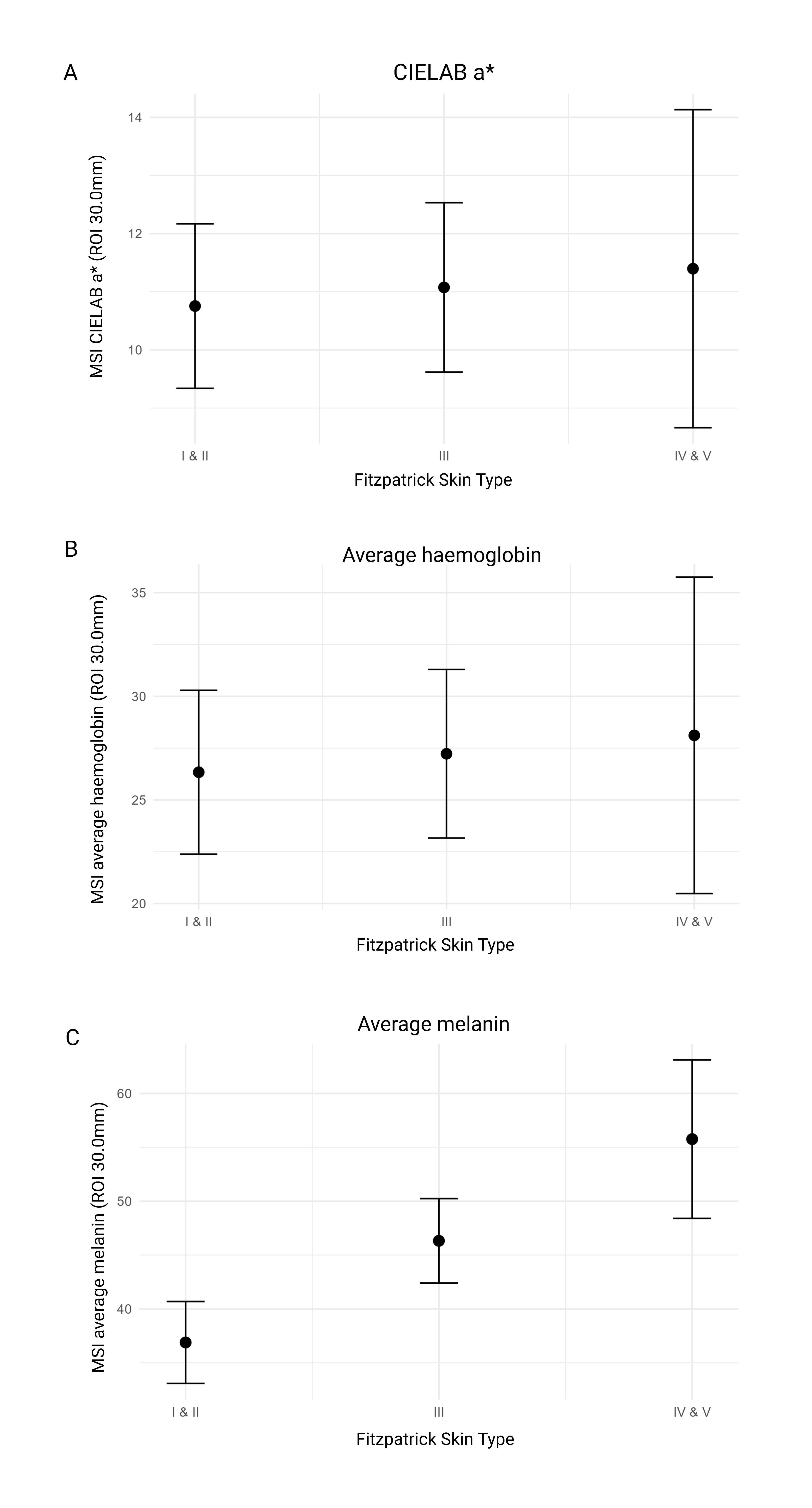

Supplement: Supplementary file 5 — Figure S5: Skin colour related parameters measured by multispectral imaging, stratified by Fitzpatrick skin type. (A) Mean CIELAB a⁎ values, (B) mean average haemoglobin levels, and (C) mean average melanin levels measured within a 30.0 mm region of interest (ROI) using multispectral imaging. Data are shown for Fitzpatrick skin types I–II, III, and IV–V. Black dots represent group means, with error bars indicating variability within each group (mean ± SD). An increasing trend is observed across Fitzpatrick skin types, most prominently for average melanin. [file EXD-35-e70236-s004.jpeg]
