# Supplementary material for: Clinical Validation of Imaging Biomarkers in Mycosis Fungoides
Source: Exp Dermatol. 2026 Mar 11;35(3):e70236. doi: 10.1111/exd.70236 (PMC12977146; doi:10.1111/exd.70236)
Supplement: Supplementary file 3 — Figure S3: Burden questionnaire for assessing patient acceptability. Burden questionnaire used to evaluate patient acceptability of various skin assessment methods. The questionnaire includes subjective burden ratings for invasive and non‐invasive skin measurements, as well as procedures conducted at home. Patients were asked to indicate their perceived burden on a visual analogue scale ranging from ‘not burdensome at all’ to ‘most burdensome ever’. [file EXD-35-e70236-s005.docx]

*Translated from original in Dutch to English*

**EXPERIENCE QUESTIONNAIRE**

We would like to ask you to answer the following statements. We ask you to indicate how burdensome you experienced each test by marking a line between two extremes: ‘not burdensome at all’ to ‘the most burdensome ever.’ The ‘burden’ of a test can be influenced by factors such as experiencing pain, discomfort, or having to sit still with legs spread for an extended period during a test. In short, anything you personally consider ‘burdensome’ can be taken into account in your response.

Beforehand, we explained the name of each test. If you no longer remember which test is being referred to in a question, you can always ask the researcher(s). If you would like to specify what you found particularly burdensome about any of the tests, you can do so in the free-text section at the end of the questionnaire.

**Section 1: Invasive skin measurements**

1. The skin biopsy was:

Not burdensome at all __________________________________________________ The most burdensome ever

2. The induction of a blister was:

Not burdensome at all __________________________________________________ The most burdensome ever

**Section 2: Non-invasive skin measurements**

3. Taking photos of the skin (2D, 3D, and 360-degree photos) was:

Not burdensome at all __________________________________________________ The most burdensome ever

4. The swab test (wet cotton) was:

Not burdensome at all __________________________________________________ The most burdensome ever

5. The blood flow measurement of the skin (Laser Speckle Contrast Imaging) was:

Not burdensome at all __________________________________________________ The most burdensome ever

6. The measurement using tape stripping (special 9 adhesive strips) on the skin was:

Not burdensome at all __________________________________________________ The most burdensome ever

7. The measurement using Optical Coherence Tomography (OCT, skin thickness measurement) was:

Not burdensome at all __________________________________________________ The most burdensome ever

8. The measurement using Thermography (skin temperature measurement) was:

Not burdensome at all __________________________________________________ The most burdensome ever

9. The transdermal analysis patch (TAP, the skin analysis plaster) was:

Not burdensome at all __________________________________________________ The most burdensome ever

10. The skin moisture measurement (TEWL, trans-epidermal water loss) was:

Not burdensome at all __________________________________________________ The most burdensome ever

**Section 3: Measurements and procedures conducted at home**

11. Collecting stool samples and sending them to CHDR was:

Not burdensome at all __________________________________________________ The most burdensome ever

12. Filling in the questionnaires via the app was:

Not burdensome at all __________________________________________________ The most burdensome ever

13. Wearing the Apple Watch at night to monitor scratching behaviour was:

Not burdensome at all __________________________________________________ The most burdensome ever

**Section 4: Remarks**
