# Supplementary material for: Clinical Validation of Imaging Biomarkers in Mycosis Fungoides
Source: Exp Dermatol. 2026 Mar 11;35(3):e70236. doi: 10.1111/exd.70236 (PMC12977146; doi:10.1111/exd.70236)
Supplement: Supplementary file 6 — Table S1: Inclusion criteria. Specific criteria required for study participation. [file EXD-35-e70236-s007.docx]

**Supplementary Table 1. Inclusion criteria**

| **DISCOVERY COHORT** | **CONFIRMATION COHORT** | **HEALTHY CONTROLS** |
| --- | --- | --- |
| 1. Able to understand and provide a written informed consent prior to any study procedures. | 1. Signed informed consent prior to any study-mandated procedure. | 1. Signed informed consent prior to any study-mandated procedure. |
| 2. Male or female subjects, 18 years or older. | 2. Male or female subjects, 18 to 75 years of age, inclusive at screening; in general, stable good health as per judgement of the investigator based upon the results of a medical history, physical examination, vital signs, ECG and laboratory assessments performed at screening. Repeated laboratory testing may be performed at the discretion of the clinical investigators. | 2. Male or female subjects, 18 to 75 years of age, inclusive at screening; in general, stable good health as per judgement of the investigator based upon the results of a medical history, physical examination, vital signs, ECG and laboratory assessments performed at screening. Repeated laboratory testing may be performed at the discretion of the clinical investigators. |
| 3. A confirmed diagnosis of CTCL (MF type or SS type) and stage classification via histology or clinico-histopathological correlation. | 3. Body mass index (BMI) ≥ 18.0 and ≤ 40.0 kg/m2; during COVID-19 pandemic only ≥ 18.0 and ≤ 33.0 kg/m2 | 3. Body mass index (BMI) ≥ 18.0 and ≤ 40.0 kg/m2; during COVID-19 pandemic only ≥ 18.0 and ≤ 33.0 kg/m2 |
| 4. For the stage IA-IIA CTCL patients: at least one patch and/or one plaque lesion are present, with at least one dimension with a diameter of ≥3 cm. | 4. At least one patch and/or plaque lesion present, with at least one dimension with a diameter of ≥ 6cm. | 4. No clinically significant skin disease as judged by the investigator. |
| 5. For the stage IIB and higher classified (tumour) CTCL patients: at least one tumour is present, with at least one dimension with a diameter of ≥1.5 cm. | 5. Confirmed MF-diagnosis (stage 1a/1b) by histology (or clinico-histopathological correlation) within the last 10 years. | 5. No history of hypertrophic scarring or keloid. |
|  | 6. Willing and able to washout any topical treatment for MF (at least 2 weeks) and any systemic treatment for MF (at least 4 weeks) prior to Day 1, resulting in a washout of 8 weeks for topical treatment and 10 weeks for disease-related systemic treatment prior to the first dosing day (day 43). | 6. Subject is willing to refrain from extensively washing (including bathing, swimming, showering and excessive sweating) the skin 4 hours before every study visit. |
|  | 7. No previous use of chlormethine gel (Ledaga) in the past two years. | 7. Subject is willing and able to washout and withhold any topical treatment (prescription and over the counter products) in the research area (if possible matched location to most common location of target lesions of the CTCL group, and otherwise 100cm2 on the lower back) for 2 weeks prior to day 1. |
|  | 8. Subject is willing and able to washout (topical and oral) antibiotic therapy for 14 days prior to day 1. | 8. Subject is willing to refrain from application of any topical product (e.g. ointments, crème or washing lotions) on the skin 24 hours prior to every study visit day. |
|  | 9. Subject is willing to refrain from extensively washing (including bathing, swimming, showering and excessive sweating) the skin 6 hours before every study visit day and up to 2 hours after application of the treatment gel. | 9. Subject is willing and able to washout (topical and oral) antibiotic therapy for 14 days prior to Day 1. |
|  | 10. Subject is willing to use effective contraception during the study if subject is male or women of childbearing potential, for up to 90 days after the last dose of study treatment. | 10. Subject is willing to use effective contraception from screening until EOS if subject is male or women of childbearing potential. |
|  | 11. Male subjects must be willing to withhold from any sperm donation during the study and up to 90 days after the last dose of study treatment. | 11. Subject has the ability to communicate well with the investigator in the Dutch language and is willing to comply with the study requirements. |
