# Supplementary material for: Clinical Validation of Imaging Biomarkers in Mycosis Fungoides
Source: Exp Dermatol. 2026 Mar 11;35(3):e70236. doi: 10.1111/exd.70236 (PMC12977146; doi:10.1111/exd.70236)
Supplement: Supplementary file 7 — Table S2: Exclusion criteria. Criteria that disqualify individuals from participating in the study. [file EXD-35-e70236-s001.docx]

**Supplementary Table 2. Exclusion criteria**

| **DISCOVERY COHORT** | **CONFIRMATION COHORT** | **HEALTHY CONTROLS** |
| --- | --- | --- |
| 1. Use of topical antibiotic (on selected target lesions) and/or oral antibiotic therapy in the previous 14 days before the visit. | 1. History of immunological abnormality (e.g., immune suppression) that may interfere with study objectives, in the opinion of the investigator. | 1. History of immunological abnormality (e.g., immune suppression) that may interfere with study objectives, in the opinion of the investigator. |
| 2. Clinically significant skin disease on the selected lesions, other than CTCL or CTCL associated secondary impetiginisation, as judged by the investigator. | 2. The use of systemic antibiotic therapy for >2 months the past 12 months. | 2. The use of systemic antibiotic therapy for >2 months the past 12 months. |
| 3. Ongoing active skin infection, other than secondary impetiginized CTCL lesions. | 3. The use of any oral/systemic medication (e.g. immunomodulatory, immunosuppressive) within 28 days prior to Day 1, if the investigator judges that it may interfere with the study objectives. | 3. The use of any oral/systemic medication (e.g. immunomodulatory, immunosuppressive) within 28 days prior to Day 1, if the investigator judges that it may interfere with the study objectives. |
| 4. Treatment of selected target CTCL lesions with radiotherapy within 8 weeks prior to Day 1. | 4. Positive hepatitis B surface antigen (HBsAg), hepatitis C antibody (HCV ab), or human immunodeficiency virus antibody (HIV ab) at screening. | 4. Positive hepatitis B surface antigen (HBsAg), hepatitis C antibody (HCV ab), or human immunodeficiency virus antibody (HIV ab) at screening. |
| 5. Any other clinical condition that may preclude participation in the study as judged by the investigator. | 5. Participation in an investigational drug study within 3 months prior to screening or more than 4 times a year. | 5. Participation in an investigational drug study within 3 months prior to screening or more than 4 times a year. |
|  | 6. Loss or donation of blood over 500mL within three months prior to screening. | 6. Loss or donation of blood over 500mL within three months prior to screening. |
|  | 7. History of alcohol consumption exceeding 5 standard drinks per day on average within 3 months of screening. Alcohol consumption will be prohibited from at least 24 hours preceding each study visit. | 7. History of alcohol consumption exceeding 5 standard drinks per day on average within 3 months of screening. Alcohol consumption will be prohibited from at least 24 hours preceding each study visit. |
|  | 8. Positive urine test for drugs or history of abuse at screening or pre-dose. Urine drug test may be repeated at the discretion of the investigator. | 8. Positive urine test for drugs or history of abuse at screening or pre-dose. Urine drug test may be repeated at the discretion of the investigator. |
|  | 9. Pregnant, a positive pregnancy test, intending to become pregnant, or breastfeeding. | 9. Pregnant, a positive pregnancy test, intending to become pregnant, or breastfeeding. |
|  | 10. Any other known factor, condition, or disease that might interfere with study conduct or interpretation. | 10. Any other known factor, condition, or disease that might interfere with study conduct or interpretation. |
|  | 11. Have any current relevant skin infections/disease in the treatment area other than the observational disease (mycosis fungoides), inclusively, but not limited to atopic dermatitis, psoriasis vulgaris, dermatomycosis and other skin malignancies. |  |
|  | 12. Having received treatments for MF or any other disease within the following intervals prior to the start of the study (The use of topical emollients is allowed during the study. For target lesions it is allowed up to 24h before every study visit day):  a. < 2 weeks for topical treatment, e.g. corticosteroids, retinoids, vitamin D analogs  b. <4 weeks for phototherapy, e.g. UVB, PUVA, PDT  c. <4 weeks for non-biologic systemic treatment, e.g. retinoids, methotrexate  d. <6 weeks for peginterferon alfa-2a  e. <8 weeks for radiotherapy or surgery in the treatment area  f. <3 months for any systemic chemotherapeutical treatment |  |
|  | 13. Known hypersensitivity to chlormethine gel or its excipients. |  |
