# Supplementary material for: Clinical Validation of Imaging Biomarkers in Mycosis Fungoides
Source: Exp Dermatol. 2026 Mar 11;35(3):e70236. doi: 10.1111/exd.70236 (PMC12977146; doi:10.1111/exd.70236)
Supplement: Supplementary file 8 — Table S3: Outcomes of multispectral imaging as a candidate imaging biomarker to quantify CAILS parameters. Outcomes of multispectral imaging as a potential imaging biomarker for assessing Composite Assessment of Index Lesion Severity (CAILS) parameters. It presents the differences (Δ) and confidence intervals (CI) for comparisons between lesional skin and healthy controls, as well as lesional and non‐lesional skin. Statistical significance is indicated by p‐values, with significant results highlighted. Colour coding: Green indicates statistically significant results; red indicates non‐significant results. Abbreviations: CI, confidence intervals; CAILS, Composite Assessment of Index Lesion Severity; MSI, multispectral imaging. [file EXD-35-e70236-s002.docx]

**Supplementary Table 3. Outcomes of multispectral imaging as a candidate imaging biomarker to quantify CAILS parameters**

|  | **LESIONAL VS HEALTHY CONTROLS** | | | **LESIONAL VS NON-LESIONAL** | | |
| --- | --- | --- | --- | --- | --- | --- |
|  | **Δ** | **CI** | **p-value** | **Δ** | **CI** | **p-value** |
| **MSI CIELAB a*** | | | | | | |
| Patch | 3.46 | 1.32-5.60 | <0.005 | 2.52 | 0.90-4.13 | <0.005 |
| Plaque | 5.43 | 2.06-8.80 | <0.005 | 4.45 | 1.45-7.52 | <0.0005 |
| Tumor | 6.97 | 3.63-10.31 | <0.0001 | 6.03 | 2.99-9.06 | <0.0001 |
| All | 5.07 | 3.04-7.11 | <0.0001 | 4.13 | 2.72-5.54 | <0.0001 |
| **MSI** **average haemoglobin** | | | | | | |
| Patch | 9.53 | 3.74-15.31 | <0.005 | 8.27 | 3.72-12.81 | <0.0001 |
| Plaque | 17.25 | 6.25-28.24 | <0.005 | 15.98 | 5.75-26.22 | <0.0001 |
| Tumor | 24.04 | 15.04-33.04 | <0.0001 | 22.78 | 14.47-31.09 | <0.0001 |
| All | 15.32 | 9.64-21.00 | <0.0005 | 14.06 | 9.87-18.25 | <0.0001 |
| **MSI average melanin** | | | | | | |
| Patch | 4.94 | 0.13-9.75 | <0.05 | 1.32 | 2.46-5.11 | 0.56 |
| Plaque | 8.68 | 1.64-15.73 | <0.05 | 5.07 | -1.28-11.42 | 0.08 |
| Tumor | 13.43 | 4.99-21.88 | <0.005 | 9.82 | 1.87-17.76 | <0.01 |
| All | 7.94 | 3.27-12.60 | <0.005 | 4.32 | 0.89-7.75 | <0.05 |
| **MSI individual topology angle** | | | | | | |
| Patch | 9.97 | 1.14-18.79 | <0.05 | 4.26 | 1.42-18.05 | <0.05 |
| Plaque | 23.13 | 9.76-36.51 | <0.005 | 17.44 | 2.55-29.74 | <0.05 |
| Tumor | 38.00 | 11.07-64.83 | <0.001 | 32.25 | -6.13-53.93 | 0.07 |
| All | 19.02 | 9.82-28.22 | <0.005 | 13.32 | 6.32-20.32 | <0.0005 |
| **MSI maximum elevation** | | | | | | |
| Patch | 0.11 | 0.01-0.20 | 0.05 | 0.10 | 0.02-0.18 | <0.005 |
| Plaque | 0.11 | 0.002-0.22 | <0.05 | 0.11 | 0.01-0.20 | <0.005 |
| Tumor | 0.34 | 0.20-0.49 | <0.0001 | 0.34 | 0.20-0.48 | <0.0001 |
| All | 0.14 | 0.06-0.21 | <0.05 | 0.13 | 0.07-0.19 | <0.0001 |
| **MSI average roughness** | | | | | | |
| Patch | 9.45 | 4.89-14.02 | <0.001 | 7.35 | 3. 14-11.56 | <0.0001 |
| Plaque | 4.42 | 0.18-8.67 | <0.05 | 2.32 | -1.70-6.35 | 0.12 |
| Tumor | 8.54 | 0.42-16.66 | <0.01 | 6.44 | -1.61-14.49 | <0.005 |
| All | 7.45 | 4.44-10.47 | <0.005 | 5.35 | 2.80-7.90 | <0.0001 |
